# Bibliographical Notices

**Published:** 1847-03

**Authors:** 


### BIBLIOGRAPHICAL NOTICES.

SYDENHAM SOCIETY'S WORKS:—*The works of William Hewson, F. R. S., edited with an Introduction and Notes*, by GEORGE GULLIVER, F. R. S., Surgeon in the Royal Regiment of Horse Guards. 8vo. pp. 360. London, 1846.

The works of Hewson, which form the first volume issued by the Sydenham Society for the year 1846, ought to be especially interesting to the American reader. Hewson himself died at the early age of 34, and his widow with her two sons and daughter, were induced to come to this country on the recommendation of Dr. Franklin, between whom, Hewson and his wife, great intimacy existed. Hewson's Experimental Inquiries into the Lymphatic System, were indeed dedicated to Franklin, "by his much obliged and most obedient humble servant."

No one at all acquainted with the progress of physiology, especially as regards the blood and the lymphatics, is unaware of the merit of Hewson as a physiologist. Still, as Mr. Gulliver remarks, "his writings have been unjustly neglected," and are now so scarce that a complete copy of them is not to be found in the store of any London bookseller, nor even in some of the best libraries, as that of the British museum." Hence, the Sydenham Society have done well to make them a part of their publications, and every one will accord with the editor, that "they will be both an acceptable present to physiological literature, and a just tribute of respect to his memory."

Of the private life of Hewson but little, it seems, is known. He was born at Hexham, in Northumberland, England, on the 14th of November, O. S., 1739; where he received the rudiments of his education at the grammar school. His father was a surgeon-apothecary in the place, and much respected. With him young Hewson acquired his first medical knowledge; but being ambitious to augment it, he placed himself first under an eminent surgeon in Newcastle, Mr. Lambert, and afterwards resided for some time in London, Edinburgh, and Paris. In the autumn of 1759, he went to London, lodged with John Hunter, and attended the lectures of Dr. William Hunter. His diligence and skill soon recommended him to the favourable notice of the Hunters, and when John went abroad as surgeon with the army, early in 1761, he left to Hewson the charge of instructing the other pupils in the dissecting room. Hewson entered himself also as a pupil at Guy's and St. Thomas's Hospitals, and went to Edinburgh, where he studied until the winter of 1762, when he returned to London, entered into partnership with Dr. Hunter, gave some lectures, and had a share of the profits. In the summer of 1765, he went to France, but returned to London in time for the anatomical lectures. In 1768, on the sea coast of Sussex, he made sundry experiments on fish; and his papers on the lymphatic system of oviparous vertebrate animals were laid before the Royal Society during the following winter, when he was made a fellow of the Society. To his recommendatory certificate were attached, amongst others, the names of Sir John Pringle, Dr. William Hunter, and Dr. Franklin.

In 1769, Dr. Hunter finished his well known building in Windmill street, where Hewson had a small apartment assigned him. They continued the lectures in partnership, dividing the profits equally—Hewson giving more of the lectures than he had formerly done. The Windmill street school is no longer in existence, but—as Mr. Gulliver remarks—"it will be preserved from oblivion by the names of the eminent men who lectured there. Among these the future historian of anatomy and physiology will have to commemorate William Hunter, Hewson, Cruikshank, Sheldon, Baillie, Brodie, Charles Bell." Mr. Gulliver might have added James Wilson—a man of decided, but modest merit, who was much respected by all who knew him, and whose name has been signalized in the "Muscle of Wilson."

In 1770, after his marriage, Hewson took a house near Dr. Hunter, in association with whom he continued to lecture during the winter of 1771. The connexion was soon after dissolved; and in 1772, Hewson began to lecture independently in Craven street, where he had built a theatre adjoining a house which he had destined for the future residence of his family. Before he began this course of anatomy, he gave a lecture on the uses of the spleen and thymus, to which he invited many men of science. His success in his first course of lectures was so great; that he had more than half the number of pupils that Dr. Hunter and he had conjointly.

Early in 1774, he had every reason to be satisfied with his position. "In viewing the situation of our associate at this period," says Dr. Lettsom—somewhat grandiloquently,—"the most gratifying prospects presented themselves, where genius and industry were rewarded with success, and domestic amities with felicity. The theatre in which he delivered his lectures and expounded his doctrines, was crowded with men of science, as well as with pupils, to listen to a youth grown sage by experimental researches." "In short," adds Mr. Gulliver, "Hewson was now surrounded by the blessings of life. He had a kind and just wife, who had borne him two lovely sons; his favorite sister lived with him; his success in teaching was no longer doubtful, and his practice in surgery and midwifery had so much increased as to give him the fairest prospect of providing well for his family. But this happiness was soon to end. He was seized with a fever occasioned by a wound received in dissection, which proved fatal on May-day 1774, after a short illness, in the thirty-fifth year of his age."

His wife, whose maiden name was Mary Stevenson,—and who appears to have been a most exemplary woman,—had been upon terms of the warmest friendship with Dr. Franklin, from the age of eighteen. She was the daughter of a lady with whom the Doctor resided during the fifteen years he passed in London. Miss Stevenson lived mostly in the country with her aunt, Mrs. Tickell, and when Hewson proposed marriage to her, she consulted Franklin on the subject. The winter of 1783–84 she spent with Franklin at Passy. He had taken especial care in the direction of her studies, and some of his best letters on philosophical subjects were addressed to her. After she lost her mother in 1783, he frequently expressed a desire that she should become his neighbour in America; and in 1786, she proceeded with her children to Philadelphia, where she lived until 1792, and then returned to Bristol, Pennsylvania, where her eldest son William had established himself, and where "she closed a well spent life on the 14th of October, 1795." "Her second son, Thomas Tickell Hewson," adds Mr. Gulliver, "who was an eminent physician, and her daughter, Mrs. Caldwell, were both living at Philadelphia in 1837. Her son William died at Vera Cruz in 1832. In the hope that some further observations might be obtained from America concerning Mr. Hewson and his descendants, the printing of this sheet has been long delayed, but my inquiries have only elicited, that his son, Dr. Hewson, is at present the respected President of the College of Physicians at Philadelphia."

Mrs. Caldwell, we would inform Mr. Gulliver, is living; and we are happy to add that the surviving son of Hewson still pursues the career of an honorable, distinguished, but unobtrusive practitioner, esteemed by all who know him, and universally and justly regarded, from a long career of honorable service, as the patriarchial head of the profession in Philadelphia,—the worthy representative of the past and the present. Well does he merit the position of President of the College of Physicians, or of any other body to which the suffrages of his medical brethren may call him!

The works of Hewson are in three parts. *Part first* treats of the properties of the blood. *Part second,* of the lymphatic system; and *Part third,* of the red particles of the blood; with some detached papers. It contains also eight copperplates, and an excellent engraved likeness of Hewson from a mezzotint in the possession of Mr. John Quekett, which is probably the same as that spoken of by Dr. Franklin, in a letter he wrote to Mrs. Hewson from Passy, in 1782;—"I forget whether I ever acknowledged the receipt of the prints of Mr. Hewson. I have one of them framed in my study. I think it very like."

The editor Mr. Gulliver, is well known for his translation of Gerber, and for his microscopical researches. He has attempted to place the matters to which his notes relate on a level with the present state of knowledge, and in this he has amply succeeded. Both in them, and in the introduction, the historical method has generally been kept in view, "because it is useful and pleasing, in a work of this nature, to mark the footsteps of the science and the names of its cultivators." The labour he has bestowed upon the subject has been great, and the results are most satisfactory. On the whole, the profession owe their thanks to the Sydenham Society for having placed in their possession—and in so beautiful a form — the works of one of the most distinguished of British physiologists.

---

*The Principles and Practice of Ophthalmic Medicine and Surgery.* By T. Wharton Jones, F. R. S., Lecturer on Anatomy, Physiology and Pathology at the Charing Cross Hospital, etc., etc. *With one hundred and two Illustrations.* Edited by Isaac Hays, M. D., Surgeon to Wills Hospital, etc. 8vo. pp. 509. Lea & Blanchard. Philadelphia, 1847.

The author of this work is well known to the profession by numerous and valuable contributions, which have appeared in the medical journals of the British Metropolis within the last few years, especially on Physiological and Pathological subjects. All his writings are distinguished by great precision and clearness as well as terseness of style, and the production before us is well entitled to this praise.

"To produce a work on Diseases of the Eye," says the author in his preface, "which should serve at once as a text-book for students and as a book of reference for practitioners, has been the great aim of the author in composing this manual. Accordingly, besides carefully discussing the principles, he has laboured to give such a practical exposition of the subject as will be found available at the bedside of the patient, and in the operating room. At the same time, he has not neglected the opportunity which the subject offers, of illustrating the general doctrines of pathology, especially those of inflammation." The author furthermore mentions "that he has incorporated in the present volume the various contributions to ophthalmic medicine and surgery which he has made, some anonymously, in the course of the last fifteen years, and also that he has freely availed himself of the information contained in the principal works, British and foreign, on the subject."

On the subject of inflammation, affecting the various tissues of the eye, the author has treated at considerable length, and certainly with great clearness. As many of the most important diseases of this delicate organ consist either in inflammation or its consequences, and as all operations upon it must be regulated by the kind and degree of inflammation expected to follow, he justly regards an accurate knowledge of it as the master-key to the whole subject. This part of the work, therefore, is particularly rich and instructive for the young practitioner; indeed the work throughout is one of very great merit, and eminently worthy of the confidence of the profession. "The author having fully posted it up to the knowledge of the day, the Editor's task," to use his own language, "has been a light one. He has restricted himself merely to the narration of the results of his own experience in a few instances in which they differed from those of the author."

The pictorial illustrations are valuable, but we cannot say that the execution of all of them is equal to what we are accustomed to see from the press of the same eminent American publishing house.

*Materia Medica and Therapeutics, including the Preparations of the Pharmacopœias of London, Edinburgh, Dublin, and [of the United States,] with many new medicines.* By J. Forbes Royle, M. D., F. R. S., late of the Medical Staff of the Bengal Army, Member of the Medical and Chirurgical Society of London, of the Medical and Physical Society of Calcutta, and ,the Royal Medical Society of Edinburgh, &c., Professor of Materia Medica and Therapeutics, King's College, London. Edited by Joseph Carson, M. D., Professor of Materia Medica in the Philadelphia College of Pharmacy, Member of the American Philosophical Society, &c., &c., with ninety eight illustrations. 8vo. pp. 689. Philadelphia, 1847. Lea & Blanchard.

Of the various works that have from time to time appeared on materia medica on the plan of the one before us, there is none more deserving of commendation. From the examination which we have given, accuracy and perspicuity seem to characterize it throughout, and as a book of reference to the student of medicine, and especially of pharmacy in its applications to medicine, none could be better. But arranged as the lectures are in our medical schools, with sessions of a duration of not longer than four months each, it would manifestly be impossible for a professor on materia medica and therapeutics to embrace the relations which drugs bear to other subjects than medicine proper; and hence the best constant accompaniment to a course of lectures on materia medica and therapeutics in our schools is one that treats of the articles as medicines chiefly. In this relation we would not place Dr. Royle's work in the very first rank, to which position it is unquestionably entitled amongst treatises which are more adapted perhaps for the pharmaceutist than the physician.

From the station, which Dr. Royle occupied in the East India service, he was enabled to collect a vast amount of information in regard to the natural and commercial history of various drugs, on which but little precise knowledge was possessed previously; and every future work on materia medica and pharmacy—every new edition of dispensatories—must be esteemed imperfect, unless it shall have culled extensively from the valuable materials contained in his pages. Dr. Royle adopts the natural arrangement, and treats, in succession, of the operations of pharmacy, pharmaceutical chemistry, mineral materia medica, vegetable materia medica, medicinal plants from ranunculaceæ to fungi; products of fermentation; animal materia medica from porifera to mammalia,—concluding with remarks on a physiological and therapeutical arrangement of the materia medica.

We think that every one who can afford it should possess this excellent work, the value of which has been greatly enhanced by the additions of Dr. Carson, than whom no one is more competent to estimate it correctly, and to make such additions as may adapt it for American service.

It is well "got up," and the wood cuts are better executed than in any work of the kind that has issued from the American press. The cause why certain plants have been selected rather than others is not apparent, but the author, we doubt not, could assign one that is satisfactory. Belladonna, Hyoscyamus and Stramonium, for example, have xylographic illlustrations, whilst Digitalis, Tabacum, Rheum, Camphora, and numerous others are neglected. The number of illustrations mentioned on the title page shows, indeed, that a large proportion of the plants must go unrepresented.

---

*Lecture Introductory to the Course on the Theory and Practice of Medicine, in the Medical Department of Pennsylvania College. Session* 1846–47. By William Darrach, M. D.

The excellent temper and good feeling exhibited in this lecture of the amiable Professor is in strong contrast with the ebullitions of spleen and mortified vanity, exhibited on like occasions by some grey-headed occupants of chairs in older and more pretending institutions. For the edification of such, we copy the opening paragraph.

"The topic, which I have been induced to select as an introductory lecture, is Life. Before I discuss it, permit me to tender you the usual, and, at the same time, most heart-felt welcome to all our Philadelphia Medical schools. Hitherto, we have been the youngest of them; and now there is yet a younger—the Franklin Medical College of Philadelphia. Prosperity to her laudable efforts! Long life to her! In her name and our own we welcome you. In that of our elder sister, the Jefferson College, in her beautifully remodelled edifice; in that of the University of Pennsylvania, the venerable and beloved mother of us all; in the name also of the departed and never to be forgotten worthies, Rush, Wistar, Barton and Physick, patriarchs of American medicine—and higher yet, we welcome you in HIS Name, the Source of life, in whom we live."

Such sentiments disarm criticism, even when invited to the task by assailable doctrines and inconclusive reasoning.

